# Bilateral stimulation: differential effects in EEG and peripheral physiology

**DOI:** 10.1192/bjo.2025.10887

**Published:** 2025-11-13

**Authors:** Markus Stingl, Eva Schäflein, Derek Spieler, Martina Henn, Bernd Hanewald, Martin Sack

**Affiliations:** Centre for Psychiatry and Psychotherapy, Justus-Liebig-University Giessen, Giessen, Germany; Department of Psychosomatic Medicine and Psychotherapy, University Hospital Rechts der Isar, Technical University Munich, Munich, Germany; Department of Psychotherapy and Psychosomatic Medicine, Faculty of Medicine, Technical University Dresden, Dresden, Germany; Department of Psychosomatic Medicine and Psychotherapy, University Hospital Erlangen, Friedrich-Alexander-University of Erlangen-Nuremberg (FAU), Erlangen, Germany; Department of Psychosomatic Medicine and Psychotherapy, Medical Centre, University of Freiburg, Freiburg im Breisgau, Germany

**Keywords:** PTSD, bilateral stimulation, eye movements, EEG, impedance cardiography

## Abstract

**Background:**

Bilateral sensory stimulation (BLS), such as eye movements or alternating tactile stimulation, is a key component of Eye Movement Desensitisation and Reprocessing (EMDR), a recommended treatment for post-traumatic stress disorder (PTSD). However, the neurophysiological mechanisms underlying BLS remain poorly understood.

**Aims:**

This study examined the physiological effects of visual and tactile BLS on frontal electroencephalography (EEG) activity and autonomic arousal in patients with PTSD and healthy controls, by varying the type of stimulation in different emotional stimuli.

**Method:**

Twenty female PTSD patients and twenty matched healthy controls participated in a counterbalanced, within-subjects design. Participants recalled a subjectively stressful or neutral event while receiving visual or tactile BLS. Frontal EEG and peripheral psychophysiological measures were recorded before and after stimulation. Data were analysed using mixed model analysis to examine the effects of stimulation type, memory condition and group.

**Results:**

Both visual and tactile BLS significantly increased the total power of frontal EEG and decreased spectral edge frequency and peripheral physiological activation. These effects were consistent between the groups and memory conditions.

**Conclusions:**

BLS, regardless of visual or tactile modality or emotional memory content, is associated with increased frontal EEG activity and reduced autonomic arousal. These findings support the hypothesis that BLS facilitates top-down cortical regulation, potentially aiding emotional processing in EMDR by using an inherent mechanism to promote psychological recovery. More research is needed to clarify the neural mechanisms and clinical implications.

Post-traumatic stress disorder (PTSD) is a generalised affect arousal dysregulation characterised by symptoms of anxiety and psychophysiological hyperarousal. The lack of top-down control of limbic activation by the prefrontal cortical structures has been repeatedly demonstrated in cases of PTSD and to a less relevant extent in other anxiety disorders.^1–3^ In contrast, increased activation of the prefrontal cortical areas has been associated with an enhanced capacity for emotional self-regulation.^1^

Eye Movement Desensitisation and Reprocessing (EMDR) is a first-line, exposure-based treatment for PTSD that directly targets the processing of traumatic memories. This is achieved by activating the memory while simultaneously applying bilateral stimulation, facilitating its integration. As a result, the memory elicits less intense emotional distress and psychophysiological arousal. Consequently, EMDR improves the patient’s ability to regulate both emotional responses and physiological arousal more effectively.^4^

During EMDR exposure, the patient is asked to visually follow the hand of the therapist moving from left to right or to focus on alternating tactile stimuli (tactile or visual bilateral stimulation). The underlying mechanisms and differential effects of bilateral stimulation (BLS) about the kind of application of this integral component of EMDR are subject to ongoing scientific debate.^5^

Based on the neurobiological findings in PTSD, several studies have focused on the cerebral and physiological effects of EMDR with its BLS approach. They predominantly differ in examination methods and sample sizes.

Electroencephalography (EEG) studies monitoring changes in brain activation during BLS do not show consistent results. In a case series of three participants, no differences were found when comparing BLS with normal waking-state EEG.^6^ Subsequently, Sharpley et al investigated healthy volunteers and found no significant changes in alpha-band EEG activity associated with eye movements compared with a baseline condition.^7^

In a series of case reports from six patients with PTSD, Harper et al observed an increase in overall EEG power during stimulation with bilateral tactile stimuli.^8^ Several studies used EEG to test the hypothesis that induced eye movements during EMDR would enhance interhemispheric interaction.^9–11^ Contrary to this hypothesis, Propper and Christman^9^ and Propper et al^10^ found decreased frontal interhemispheric coherence in the gamma EEG band after stimulation with eye movements. Another study by Samara et al^11^ also found no evidence for increased hemispheric interaction.

Pagani et al^12^ studied PTSD patients during recall of traumatic events. Following the EMDR protocol, bilateral ocular stimulation significantly increased EEG activity in the frontal cortical areas. The analysis also indicated significant shifts in cerebral activation from the frontotemporal to temporo-occipital regions. In a subsequent study, the authors could confirm their findings and interpret their observations as potential indicators for a shift from a predominantly emotional processing of traumatic information to a more explicit and higher cognitive processing.^13^ Additionally, single-photon emission tomography (SPECT) studies have shown an association between successful treatment for PTSD and normalisation of frontal cerebral activity.^14–15^

Studies monitoring psychophysiological arousal during EMDR therapy with PTSD patients report a decrease in arousal at the beginning of stimulation, a decrease in heart rate and an increase in parasympathetic tone throughout the entire session.^16,17^ The speed and extent of these observed psychophysiological effects in EMDR could not be attributed solely to habituation; therefore, the authors conclude that BLS induces orientation responses with an accompanying decrease in psychophysiological arousal. Thus, BLS could enhance self-regulation capacities by reducing physiological fear responses during exposure and facilitating the input of new trauma-related adaptive information.^18^

BLS can be done in different ways. Predominantly, stimulation is induced visually, either by having the eyes follow the therapist’s hand or by using moving stimuli (e.g. dots) on light bars or screens. Another form of application is tactile BLS, which is triggered by the therapist tapping alternately on the back of the patient’s hand. This can also be done with vibrating alternating hand devices. The frequency of the stimulation varies around 1 Hz.

Several studies have investigated the effects of applying different types of BLS, and a review^19^ summarises studies examining different variants of BLS and other dual tasks. The overview of laboratory studies carried out showed that eye movements and alternative dual tasks produced similar decreases in vividness and emotionality, with the impact on vividness being stronger than on emotionality. However, eye movements yielded a marginally stronger overall vividness reduction than alternative dual tasks such as binaural stimulation, playing Tetris or counting. Only one study contrasted the effects of visually induced BLS and tactile stimulation.^20^ In this study, in contrast to tactile stimulation, the participants’ self-rated scores showed that negative memories became less negative and positive memories less positive when visually induced BLS was used. However, the condition of tactile stimulation was created by rhythmically tapping the tabletop with the index and middle finger together, which cannot be considered a BLS intervention.

With respect to the content of the processed memories, there are also heterogeneous findings. In clinical practice, BLS is used to reduce the load of distressing memories as well as to enhance and strengthen positive memories and resources. Although the effects of BLS on negative memories have been the target of many studies, examinations of the effects of BLS on neutral and/or positive memories or imaginings are sparse.

To summarise, there is evidence of BLS’s impact on cerebral activation and psychophysiological arousal. However, it remains unclear to what extent the type of BLS (tactile or eye movements) influences the observed effects and whether these effects are common or specific for patients suffering from PTSD alone. Furthermore, the question arises as to what extent BLS effects depend on the content and emotional quality of the processed material and whether these effects have an influence on treatment success or the identification of subgroups.

## Research questions

To differentiate the central and peripheral psychophysiological effects of BLS, we investigated two usually in EMDR applied stimulation methods (eye movements and tactile stimulation). Therefore, we compared the cerebral activation patterns (EEG) and peripheral physiological reactions (impedance cardiography) of patients with PTSD with healthy controls while being exposed to neutral and distressing memories.

## Method

### Participants

The experimental population consisted of 40 women, half of whom were patients and the other half were healthy controls. The patients were out-patients from a centre specialising in psychotraumatology at the Department of Psychosomatic Medicine, Klinik Rechts der Isar, Technical University Munich. Information about the objectives of the study was provided and all subjects gave their written informed consent.

PTSD patients were significantly older than healthy participants (M_patients_ = 38,3; s.d._patients_ = 8.5; M_control_ = 30, 4; s.d._control_ = 13, 1; *p* = 0.029; *F*(39) = 5.2), therefore, all statistical comparisons were controlled for age.

All patients had PTSD assessed by experienced clinicians via the PTSD module of the Structured Clinical Interview for DSM-IV (SCID-IV).^21^ Five patients reported being assault victims, three were accident survivors, one participant accidentally killed a child, six reported being victims of sexual violence and five patients experienced both sexual and physical violence.

Six patients took antidepressants and one patient additionally took an antipsychotic drug. Knowing the long-term effects of antidepressants on brain activity in terms of functional normalisation of the ‘anxiety network’ and, in particular, a reduction in overactivity of limbic structures,^27^ we decided to include these participants. Therefore, the psychiatric medication had to be stable to avoid possible short-term effects. Healthy control subjects did not take medication.

Criteria for exclusion from the study included a history of psychotic disorder, current substance abuse or other unstable psychological conditions, to prevent confrontation with traumatic reminders.

### Ethical approval

The authors assert that all procedures contributing to this work comply with the ethical standards of the relevant national and institutional committees on human experimentation and with the Declaration of Helsinki of 1975, as revised in 2013. All procedures involving human subjects/patients were approved by the Ethics Committee of Technical University Munich (approval number: 5068.11).

### Experimental design

In a preliminary stage, patients were asked to choose a traumatic memory, while healthy controls were asked to choose a personal distressing memory (when a traumatic memory was absent) with subjective distress of no more than 5 on a scale from 0 (= not distressing) to 10 (maximum distressing). A verbal report of 30 s was transcribed into individual trauma scripts. The scripts were then read to the patient to check for any inconsistencies between their memories and the recorded audio tape. The neutral scripts, which described a common everyday life situation (washing the dishes), were not individualised.

All subjects of the study participated in a single-session experiment comprising four blocks, varying the type of memory (neutral versus distressing) and BLS (eye movements versus tactile stimulation). Each block consisted of three phases lasting 30 s each: starting imagination (pre-stimulation phase), BLS and post-stimulation phase. Before and after each block, a 3-min relaxation phase was included. Patients and controls were randomised into two groups of ten subjects. To balance the sequence of stimulation, the first group started with tactile stimulation and the other group with eye movements. Eye movements were induced using a light bar looping from side to side, with 16 small red lamps (EMDR Eye Scan Unit, EMDR Equipment Europe), with an overall frequency of 1 Hz. Tactile stimulation was applied by a pair of alternately vibrating plastic pulsers (EMDR Tactile Unit Boka 9, EMDR Equipment Europe) with a frequency of 1 Hz.

### Psychophysiological assessment

The total EEG power was evaluated with a bispectral index (BIS) monitor (Aspect Medical Systems, Norwood, Massachusetts, USA; Medical Advisory Secretariat, 2004). The BIS measurement set consists of five disposable Ag/AgCl electrodes (Covidien LLC, Mansfield, Massachusetts, USA) attached to both sides of the forehead (10/20 system locations: FP1, FP2, A1, A2, plus a central reference electrode). Data were sampled with a frequency of 256 Hz and analogue bandpass filtering of 0.3–70 Hz was applied. Data were excluded if electrode impedances were above 7.5 kOhm, when artefacts were visible or if the signal quality index was <80% (SQI; quantified as the percentage of the previous 60 s of data that could be used to calculate EEG spectral variables). In the data of one participant, a single measurement point had to be excluded from the analysis due to a low SQI.

In addition to the raw EEG signals, we collected the total EEG power in the 0.5–30 Hz frequency range (TOTPOW; the sum of different frequency ranges obtained during the EEG recording) from both hemispheres and the spectral edge frequency (SEF: the frequency below which 95% of the total EEG power resides). To calculate the total bilateral frontal EEG power, we averaged TOTPOW values from the two hemispheres.

Peripheral psychophysiological activation was recorded by impedance cardiography (ICG) with the Ambulatory Monitoring System (AMS; Department of Psychophysiology, Vrije University, Amsterdam, The Netherlands) using seven disposable pre-gelled self-adhesive surface electrodes (Neuroline 720, Ambu, Ballerup, Denmark) attached to the thorax. Electrocardiogram (ECG) and impedance cardiography signals (ICG) were recorded with a sample rate of 1000 Hz. The validity and reliability of the VU-AMS system have previously been demonstrated.^30^

Interbeat intervals (IBIs) were calculated as differences between adjacent R-R signals. Changes in the sympathetic drive were measured by changes in the pre-ejection period (PEP), while the root-mean-square of successive differences (RMSSD) of successive IBIs represented changes in the parasympathetic tone. For further analysis, due to the statistical skewness of RMSSD, its natural logarithm (LNSSD) was calculated. Furthermore, the bandpass-filtered thoracic impedance allowed an estimation of respiratory frequency. For the processing of physiological data, the VU-AMS data were analysed with the Vrije Universiteit Data Analysis and Management Software (VU-DAMS) version 3.2 for Windows (VU – Ambulatory Monitoring Solutions B.V., Amsterdam, The Netherlands; see https://vu-ams.nl/downloads/). ECG data was visually checked for artefacts. No artefacts were detected, and all files were processed.

### Instruments

The Impact of Event Scale (IES) was administered to assess the severity of trauma-related symptoms.^24^ A Subjective Units of Disturbance Scale (SUD) was used to assess the subjective intensity of distress caused by traumatic memories ranging from no distress (0) to maximum distress (10).

### Data analysis

Frontal EEG power and SEF, and psychophysiological activity before and after stimulation, were compared by calculating the mean values of the indices (TOTPOW, SEF, PEP, RMSSD) in each pre-stimulation and post-stimulation phase. During stimulation, the data were not analysed to avoid artefacts resulting from eye movements. TOTPOW and SEF of the left and right hemispheres were averaged during trials and the mean values before and after stimulation were compared. We used a pre/post repeated measures design to examine differences in values in TOTPOW and SEF before and after stimulation and applied a linear mixed-effect statistical model. Fixed effects were group (patients/healthy), quality of memory (neutral/stressful), stimulation method (eye movements/tactile) and phase (pre/post). The within-subject factor was selected as the random effect. Due to the significant differences between the groups, age was included as a covariate. IES and SUD group comparisons were analysed using ANOVA. Calculations were analysed with the Statistical Package for Social Sciences (SPSS 24, IBM Inc.).

## Results

### Subjective findings

Compared to the healthy control group, PTSD patients showed significantly more emotional strain in IES (d_M_ = 32.6; *p* < 0.001; *F*(39) = 88.8) and higher subjective stress (SUD) while recalling a distressing memory (d_M_ = 1.5; *p* < 0.001; *F*(39) = 27.7).

### Effects of eye movements

When comparing the pre/post effects of eye movements, a significant increase in TOTPOW and a significant decrease in SEF were observed after eye movement stimulation. Furthermore, this type of BLS led to a decrease in autonomic activity, as implied by increased IBIs, increased heart rate variability (RMSSD) and significantly reduced respiration rates (RESP).

The psychophysiological effects observed after eye movements were the same for the factors ‘group’ (patients versus healthy people) and ‘quality of memory’ (neutral versus stressful) (Table 1).

**Table 1:** Stimulation with eye movements: comparison of psychophysiological variables (mixed model analysis)

Impedance cardiography	Group				Memory activation				Pre-/post-stimulation			
	Healthy	PTSD	Between-groups comparison (LMM)		No-trauma	Trauma	Repeated-effects comparison (LMM)		Pre	Post	Repeated-effects comparison (LMM)	
	Mean (s.d.)	Mean (s.d)	*F*(d.f.)	Sign.	Mean (s.d.)	Mean (s.d.)	*F*(d.f.)	Sign.	Mean (s.d.)	Mean (s.d.)	*F*(d.f.)	Sign.
IBI (ms)	842 (140)	787 (109)	1.9 (38.0)	n.s	812 (128)	816 (128)	1.2 (100.6)	n.s.	805 (124)	823 (131)	17.5 (100.6)	*p* < 0.001 ***
LNSSD (ms)	3.43 (0.60)	3.01 (0.70)	4.7 (37.4)	*p* = 0.036^+^	3.23 (0.67)	3.22 (0.70)	0.04 (112.9)	n.s	3.15 (0.71)	3.29 (0.64)	10.4 (112.9)	*p* = 0.002 **
PEP (ms)	69.6 (10.3)	70.4 (8.9)	0.8 (44.0)	n.s.	69.1 (10.6)	70.9 (8.5)	3.3 (87.7)	*p* = .073^+^	70.7 (9.3)	69.4 (9.9)	1.7 (87.7)	n.s.
RESP (bpm)	15.0 (2.5)	13.9 (3.0)	2.3 (37.4)	n.s.	14.3 (2.8)	14.6 (2.8)	0.75 (106.5)	n.s.	15.3 (2.9)	13.6 (2.5)	47.4 (106.5)	*p* < 0.001 ***
**Frontal qEEG**												
TOTPOW (µV^2^)	61.4 (3.9)	61.0 (3.8)	0.33 (51.0)	n.s.	61.4 (3.8)	61.0 (3.9)	0.93 (78.7)	n.s.	58.5 (2.3)	63.9 (3.2)	224 (78.7)	*p* < 0.001 ***
SEF (Hz)	19.6 (3.6)	21.4 (3.9)	3.4 (45.2)	*p* = 0.073^+^	20.3 (4.1)	20.7 (3.7)	0.86 (85.6)	n.s.	21.6 (3.0)	19.4 (4.4)	31.6 (85.6)	*p* < 0.001 ***

IBI, inter-beat interval; LMM, linear mixed models; LNSSD, (ln) root mean square of successive differences of inter-beat intervals; PEP, pre-ejection period; PTSD, post-traumatic stress disorder; qEEG, quantitative electroencephalography analysis; RESP, respiration frequency; TOTPOW, total electroencephalography power; SEF, spectral edge frequency; n.s., not significant after controlling for influences of age; +:Trend (*p* = 0.05 to 0.10), *p = 0.01 to 0.05, **p = 0.001 to 0.01, ***p < 0.001.

### Effects of tactile stimulation

When contrasting the pre/post effects of tactile stimulation, a significant increase in TOTPOW and a significant decrease in SEF within the post-stimulation phase occurred. After tactile stimulation, psychophysiological activity was reduced as suggested by the significantly increased heart rate variability (RMSSD) and significantly reduced RESP. No significant changes were noticed for IBI.

When comparing the central and peripheral physiological effects of subsequent tactile stimulation in patients with PTSD and healthy controls, no significant group effects were found. Additionally, there were no significant differences between the effects of neutral and stressful memory (Table 2).

**Table 2:** Tactile stimulation: comparison of psychophysiological variables (mixed model analysis)

Impedance cardiography	Group				Memory activation				Pre-/post-stimulation			
	Healthy	PTSD	Between-groups comparison (LMM)		No-trauma	Trauma	Repeated-effects comparison (LMM)		Pre	Post	Repeated-effects comparison (LMM)	
	Mean (s.d.)	Mean (s.d.)	*F*(d.f.)	Sign.	Mean (s.d.)	Mean (s.d.)	*F*(d.f.)	Sign.	Mean (s.d.)	Mean (s.d.)	*F*(d.f.)	Sign.
IBI (ms)	844 (139)	794 (102)	1.6 (38.0)	n.s.	821 (123)	818 (126)	0.42 (107.5)	n.s.	819 (127)	820 (123)	0.02 (107.5)	n.s.
LNSSD (ms)	3.46 (0.57)	3.03 (0.70)	5.1 (38.1)	*p* = 0.030*	3.26 (0.68)	3.24 (0.67)	0.26 (108.3)	n.s.	3.20 (0.67)	3.30 (0.67)	5.5 (108.3)	*p* = 0.021 *
PEP (ms)	69.2 (11.5)	69.7 (11.5)	0.02 (41.1)	n.s.	70.7 (9.5)	69.1 (13.1)	3.8 (85.3)	*p* = .053^+^	70.0 (11)	68.8 (12)	0.77 (85.3)	n.s.
RESP (bpm)	15.0 (2.6)	14.0 (3.1)	1.7 (37.2)	n.s.	14.5 (3.0)	14.5 (2.9)	0.10 (113.1)	n.s.	15.0 (3.0)	14.0 (2.7)	16.7 (113.1)	*p* < 0.001 ***
**Frontal qEEG**												
TOTPOW (µV^2^)	61.2 (4.3)	61.0 (4.5)	0.43 (53.3)	n.s.	61.3 (4.4)	61.0 (4.4)	0.74 (77.3)	n.s.	58.1 (2.4)	64.1 (3.9)	211 (77.3)	*p* < 0.001 ***
SEF (Hz)	19.6 (3.2)	21.1 (3.8)	2.4 (39.3)	n.s.	20.2 (3.4)	20.5 (3.6)	0.47 (88.0)	n.s.	21.2 (3.2)	19.5 (3.8)	22.7 (88.0)	*p* < 0.001 ***

IBI, inter-beat interval; LMM, linear mixed models; LNSSD, (ln) root mean square of successive differences of inter-beat intervals; PEP, pre-ejection period; PTSD, post-traumatic stress disorder; qEEG, quantitative electroencephalography analysis; RESP, respiration frequency; TOTPOW, total electroencephalography power; SEF, spectral edge frequency; n.s., not significant after controlling for influences of age; +:Trend (*p* = 0.05 to 0.10), *p = 0.01 to 0.05, **p = 0.001 to 0.01, ***p < 0.001.

**Fig. 1:**
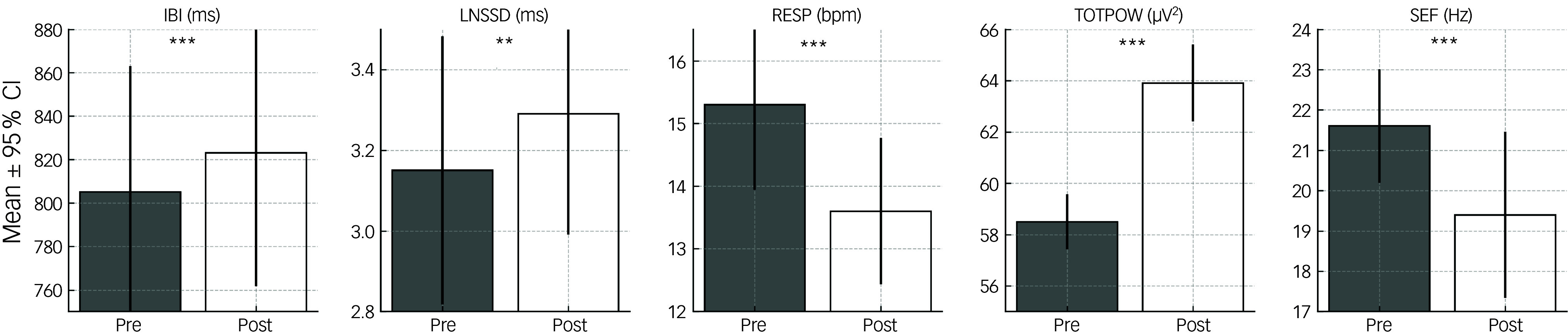
Peripheral and central effects of visual stimulation. IBI, inter-beat interval; LNSSD, (ln) root mean square of successive differences of inter-beat intervals; RESP, respiration frequency; TOTPOW, total electroencephalography power; SEF, spectral edge frequency; **p = 0.001 to 0.01, ***p < 0.001.

**Fig. 2:**
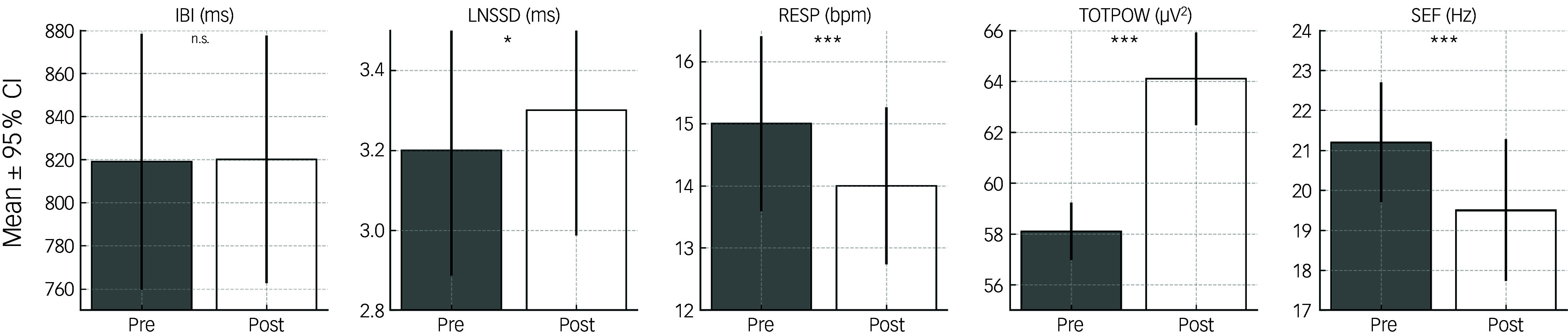
Peripheral and central effects of tactile stimulation. IBI, inter-beat interval; LNSSD, (ln) root mean square of successive differences of inter-beat intervals; RESP, respiration frequency; TOTPOW, total electroencephalography power; SEF, spectral edge frequency; *p = 0.01 to 0.05, ***p < 0.001.

Figures 1 and 2 present a summary of all significant results for visual stimulation (Fig. 1) and tactile stimulation (Fig. 2).

## Discussion

We investigated the different psychophysiological effects of two types of stimulation commonly used in EMDR interventions (eye movements and bilateral tactile stimulation). The activation patterns before and after the imagining of a distressing or a neutral memory were compared in healthy participants and patients with PTSD. Both types of BLS led to a significant increase in frontal EEG total power, a decreased SEF and decreased psychophysiological activation, representing a significant change in frontal brain activity accompanied by physiological relaxation. These effects were observed in both PTSD patients and healthy controls and were irrespective of listening to individualised distressing or neutral memories.

Clearly, our data would be in line with previous studies that describe changes in frontal EEG activation after eye movements and tactile stimulation.^7,15^ Frontal brain structures, especially the ventromedial prefrontal cortex (vmPFC), play a crucial role in the pathogenesis and maintenance of PTSD symptoms. In a meta-analysis,^25^ reduced cerebral activity is described in the frontal areas of the brain in PTSD patients compared to other anxiety disorders. More in detail, several studies have shown that the vmPFC is significantly involved in learning safety signals and differentiating between safety and fear signals,^26^ both of possible targets in PTSD treatment. Furthermore, reduced frontal neuronal activity in PTSD is associated with impaired fear extinction and frontal brain lobes are described to be involved in the difficulties of PTSD patients mediating affect and cognition.^25–29^ Beak and colleagues^30^ elaborated on the effects of bilateral visual stimulation in mice and identified neural circuits that are involved in the long-lasting attenuation of fear responses: activation of the superior colliculus through BLS could contribute to fear extinction by supporting activity in the mediodorsal thalamus and prefrontal cortex to compete with emotional activity in the amygdala. Therefore, our findings of BLS-associated increased frontal EEG total power and reduced spectral edge frequency could have implications for the treatment of patients with PTSD.

The results of decreased psychophysiological activities after BLS match with earlier studies that reported similar effects on peripheral psychophysiological activation. These studies attribute these effects to stimulation-associated orienting responses.^16,17,31^ The peripheral psychophysiological results might suggest an effect of de-arousal^32^ and relaxation during the experimental condition.^16,17,33^

Neither the emotional content (neutral or distressing) nor differences in the participants (healthy volunteers or patients suffering from PTSD) influenced the activation patterns of EEG or the peripheral psychophysiological changes observed in our study. A potential explanation for the absence of difference between neutral and distressing memories could be that the distressing memory had been defined at the beginning of the experiment. Therefore, it is possible that participants would have thought of the distressing event even when asked to think of the neutral scene, leading to a carryover effect. Another reason might be that, for ethical reasons, we decided to select a disturbing memory, which was only of medium strain. The results for patients and healthy controls could have been different if patients had thought of a more stressful traumatic event during the stimulation periods. Furthermore, an alternative explanation from an evolutionary psychology perspective, which goes beyond the specific effects of the study design, could be that the identified mechanism is generic and reflects an evolutionary adaptation to adverse circumstances.

One previous study indicates that stimulation associated with prefrontal activation represents the effects of attention to novel events.^34^ The stimulation periods (eye movements, tactile stimulation) in our study can be seen as novel events during the experiment. This may explain why the stimulation associated with EEG activation observed in our study could be relatively unspecific to the characteristics of the participants, as we found the same effects in patients with PTSD and healthy controls and could be interpreted as an effect of general activation. This is also in line with assumptions of the orienting response theory^35^ which assumes that repetitive stimuli trigger an intensified orientation reaction. The term describes a short time (fewer than 10 s) increase in attention toward new or changing stimuli of low or middle intensity, consisting of an activation of the parasympathetic system and a simultaneous activation of the sympathetic system, followed by a habituation response. Through repetitive stimulation, this first response is intensified, provoking investigatory activity in attention networks involved in the self-regulation of emotional reagibility.^36,37^ In consequence, an activity of the behavioural avoidance system is said to be reduced and changes in the narrative representation of the emotional situation are facilitated. Trauma-focusing treatments, such as EMDR, can use this mechanism to reduce the avoidance of distressing pathogenic memories.

As our study has shown, both types of stimulation (visual and tactile) have significant central and peripheral physiological effects in the same direction. In clinical practice, visual stimulation is said to be the first line way to induce BLS, but tactile stimulation can also be used in relation to the patient’s abilities, tolerance, and comfort. Studies using or contrasting tactile and visual stimulation show comparable effects^38^ while the question of underlying common or different working mechanisms remains open at this time. As only one type of BLS has been used at a time in the study as common in EMDR therapy, the results could support the combination of different BLS techniques as they are sometimes already used in practice, but suitable studies are still lacking. Initial approaches in this direction can be found in G-TEP (group traumatic episode protocol),^39^ a promising group EMDR intervention in which patients self-stimulate by visually tracking how their hands move from side to side while tapping on an elaborated worksheet. Further studies should investigate the potential benefits or additive effects of such a combination of BLS.

Our study has several limitations. The BIS Vista system does not provide precise spectral data. Therefore, its EEG values are difficult to interpret, do not allow final conclusions if an increase in TOTPOW and a decrease in SEF represent cortical activation or suppression, and not allowing source location techniques. Despite the limitations mentioned, we used this EEG technique because it is practical and gentler on already severely stressed PTSD patients.

Furthermore, the study is based on an experimental design and is not a real-life treatment study. Additionally, we did not use any measure of vividness or extent of emotionality of memories. Another major limitation is the non-availability of an eye-tracking device, so influences of involuntary eye movements on EEG measures may have been overlooked. We did not follow the entire EMDR protocol, which elaborately includes beliefs related to trauma and physical sensations, and the sample is characterised by a heterogeneous trauma history and concurrent use of medication. The duration of bilateral stimulation did not match that typically used in clinical practice, which is of greater duration and, therefore, may have implications on the magnitude of effectiveness. Furthermore, since only women were included and since a review^40^ reports sex-related differences in vulnerability and autonomic control of PTSD patients, the generalisability of our results is limited to women only.

In summary, our study identified alterations in frontal EEG and a decrease in psychophysiological activation after two different BLS methods (eye movements and tactile stimulation). These effects were observed after the imagining of neutral and distressing memories in women, both healthy and suffering from PTSD. The findings might support the assumption that stimulation with eye movements or bilateral tactile stimuli enhances cortical top-down control and therefore potentially assists in processing distressing memories. They also support the assertion that EMDR uses an inherent mechanism to promote psychological recovery. Future studies could further investigate these effects and determine their clinical relevance to treatment, especially using more precise EEG measurements, and directly compare the effects of different types of stimulation or their combination.

## Data Availability

The data that support the findings of this study are not publicly available due to privacy restrictions. The data are available from the corresponding author, M.St., upon reasonable request.
